# Our Associated Editors

**Published:** 1871-08

**Authors:** 


### PART IV.

### Editorials, Reviews, Items and News.

### OUR ASSOCIATED EDITORS.

With this issue of the COMPANION, we add to the number of our Associated Editors. These gentlemen are so well known as to make it unnessary for us to allude to them individually. Their contributions to the medical literature of the country, speak for them in the past. In the future, through our JOURNAL, we hope to introduce them again to their old friends, and to hundreds of new ones. These gentlemen enjoy great experience and possess stores of valuable information, which the profession should insist upon obtaining as its due; and we feel confident they will respond promptly and generously, as often as time and opportunity will permit, to such a demand upon them.

Dr. Robert Battey,—among the first of his profession of the State,—leads off in an article of great practical value to every physician—on enemata; a careful reading of which, will prove beneficial to young and old.

As an evidence of the appreciation of the benefits which may be expected from the co-operation of these gentlemen to the profession and the cause of medical science, we give, below, an extract from a letter of a distinguished medical gentleman of this State. Had we space, we could fill pages with extracts from letters received from gentlemen of Georgia and adjoining States, breathing the same sentiments.

